# DPYD genotype-guided dose personalisation for fluoropyrimidine-based chemotherapy prescribing in solid organ cancer patients in Australia: GeneScreen 5-FU study protocol

**DOI:** 10.1186/s12885-024-13122-8

**Published:** 2024-11-08

**Authors:** Cassandra White, Hannah Wardill, Christine Paul, Timothy Price, Christos Karapetis, Mark Nalder, Matthew E. Burge, Ann Thomas, Chris Oldmeadow, Daniel Barker, Laura C. Edney, Janet Coller, Joanne Bowen, Cheri Ostroff, Bruce Cheek, Mel Carlson, Trumaine Rankmore, Adnan Nagrial, Stephen Clarke, Lorraine Chantrill, Stephen Ackland, Rodney J. Scott

**Affiliations:** 1 University of Newcastle, College of Health, Medicine and Wellbeing, School of Medicine and Public Health, Callaghan, NSW 2308 Australia; 2 Hunter Medical Research Institute, Lot 1, Kookaburra Circuit, New Lambton Heights, NSW 2305 Australia; 3 School of Biomedicine, University of Adelaide, Adelaide, SA 5005 Australia; 4 Supportive Oncology Research Group, Precision Cancer Medicine, South Australian Health and Medical Research Institute, Adelaide, SA 5000 Australia; 5 The Queen Elizabeth Hospital and University of Adelaide, Adelaide, SA 5005 Australia; 6 Flinders University and Flinders Medical Centre, Bedford Park, SA 5042 Australia; 7 Royal Brisbane and Women’s Hospital, Brisbane, QLD 4006 Australia; 8 University of Queensland, Brisbane, QLD 4006 Australia; 9 Flinders University, College of Medicine and Public Health; Flinders Health and Medical Research Institute, Adelaide, SA 5042 Australia; 10 University of South Australia, Centre for Workplace Excellence, Adelaide, SA 5001 Australia; 11 Australasian Gastrointestinal Trials Group (Consumer Panel), Camperdown, NSW 2050 Australia; 12 Cancer Quality of Life Expert Service Team (Member Steering Committee), University of Technology Sydney, Ultimo, NSW 2007 Australia; 13 Cancer Voices New South Wales (Consumer Representative), Milsons Point, NSW 1565 Australia; 14 Western Sydney Local Health District, Westmead, NSW 2148 Australia; 15 University of Sydney, Westmead Clinical School, Westmead, NSW 2148 Australia; 16 Northern Sydney Local Health District, St. Leonards, NSW 2065 Australia; 17 University of Sydney, Northern Clinical School, St. Leonards, NSW 2065 Australia; 18 Illawarra Shoalhaven Local Health District, Wollongong, NSW 2500 Australia; 19 University of Wollongong, Wollongong, NSW 2500 Australia; 20 University of Newcastle, College of Health, Medicine and Wellbeing, School of Biomedical Science and Pharmacy, Callaghan, NSW 2308 Australia; 21 Department of Molecular Genetics, Pathology North John Hunter Hospital, New Lambton Heights, NSW 2305 Australia

**Keywords:** *DPYD*, Fluoropyrimidine, Pharmacogenomics, Dihydropyrimidine dehydrogenase, *UGT1A1*, Irinotecan

## Abstract

**Background:**

Fluoropyrimidine (FP) chemotherapies are commonly prescribed for upper and lower gastrointestinal, breast and head and neck malignancies. Over 16,000 people with cancer require FP chemotherapies *per annum* in Australia. Between 10 and 40% patients experience grade 3–4 (≥ G3) toxicities that require hospital-based management ± intensive care admission. Approximately 1% of patients die secondary to FP toxicities. Prospective screening for *DPYD* gene variants (encoding the key enzyme for FP catabolism) can identify patients at risk of ≥ G3 toxicity and allow for dose adjustment prior to first FP exposure. Evidence supports this as a cost-effective method of improving patient safety and reducing healthcare burden internationally; however, no Australian data confirms its feasibility on a large scale.

**Method:**

This investigator-led, single-arm study will determine large scale feasibility of prospective *DPYD* genotyping, confirming patient safety and cost-effectiveness within the Australian health care system. 5000 patients aged 18 years and older with solid organ cancers requiring FP chemotherapy will be consented and genotyped prior to commencing treatment, and early toxicity (within 60 days) post-FP exposure will be determined. Toxicity data for *DPYD* variant carriers who have dose adjustments will be compared to the wild-type cohort and historical cohorts of carriers who did not undergo genotyping prior to FP exposure, and prospective variant carriers who do not undergo dose-adjustment. Prevalence of the four standard *DPYD* gene variants will be confirmed in an Australian population. Additionally, health economic analysis, implementation research via semi-structured interviews of patients and clinicians, and feasibility of *UGT1A1* genotyping will be conducted.

**Discussion:**

This study will determine the prevalence of *DPYD* gene variant status in Australia and its impact on FP-induced toxicity among Australians with cancer. Feasibility and cost-effectiveness for Australian health care system will be estimated to support national roll-out of prospective *DPYD* genotyping prior to FP administration. Additionally, feasibility will be confirmed with the intention of including *UGT1A1* in future pharmacogenomic panels to aid chemotherapy prescribing.

**Trial registration:**

This trial was registered with the Australian and New Zealand Cancer Trials Registry on 13th Dec 2023, ACTRN12623001301651.

**Supplementary Information:**

The online version contains supplementary material available at 10.1186/s12885-024-13122-8.

## Background

Fluoropyrimidines (FP) are commonly prescribed and variably toxic chemotherapies. Over 16,000 adult Australians receive this chemotherapy per year for the treatment of colorectal, upper gastrointestinal, breast and head and neck cancers [1]. Globally, more than 3.5million patients receive FP chemotherapies [2]. FP is delivered either as intravenous (5-fluoruracil; 5-FU) or oral (capecitabine) preparations.

**Fig. 1:**
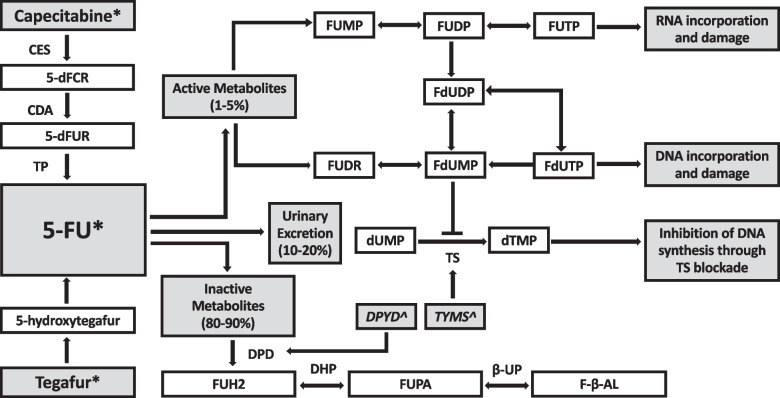
Metabolic Pathway of Fluoropyrimidines, adapted from White [8]. Legend: * Fluoropyrimidine chemotherapeutic agents, ^ Genes. Enzymes: CES; carboxyl esterase, CDA; cytodine deaminsae, TP; thymidine phosphorylase, TS; thymidylate synthase, DPD; dihydropyrimidine dehydrogenase, DHP; dihydropyrimidinase, B-UP; beta-ureidopropionase. Metabolites: 5’dFCR; 5’-deoxyfluorocytidine riboside, 5’dFUR; 5’-deoxyfluorouridine, FUH2; dihydrofluorouracil, FUPA; fluoro-beta-ureidopropionate, FBAL; fluoro-beta-alanine, FUMP; fluorouridine monophosphate, FUDP; fluorouridine diphosphate, FUTP; fluorouridine triphosphate, FUDR; fluorodeoxyuridine, FdUMP; fluorodeoxyuridine monophosphate, FdUDP; fluorodeoxyuridine diphosphate, FdUTP; fluorodeoxyuridine triphosphate, dUMP; deoxyuridine monophosphate, dTMP; deoxythymidine monophosphate. Genes: DPYD encodes DPD, TYMS encodes TS

Between 10–40% of patients who receive FP therapy develop severe and life-threatening (National Cancer Institute Common Terminology Criteria for Adverse Events, CTCAE grade 3 and 4) toxicities, and up to 1% die as a result of toxicity (CTCAE grade 5) [3–5]. Typical toxicities include diarrhoea, mucositis, haematological toxicities (predominantly neutropenia and thrombocytopenia), cardiotoxicity and palmar-plantar erythrodysesthesia (hand-foot syndrome) [6]. Between 31–69% of severe toxicities can be explained by a deficiency in the dihydropyrimidine dehydrogenase (DPD) enzyme, encoded by the *DPYD* gene (Fig. 1) [7, 8]. DPD catabolises circulating 5-FU, allowing excretion of inactive metabolites [9]. People who are DPD deficient lack the capacity to adequately catabolise circulating 5-FU, creating a supratherapeutic build-up of active drug and excessive toxicities as a result. Testing for DPD functional activity is difficult and surrogate tests such as *DPYD* genotyping have been developed to overcome this, though no test has yet been devised that can accurately identify all patients at risk of severe FP toxicities [10].

Throughout Europe, four clinically significant *DPYD* variants are increasingly screened prior to administration of FP agents, endorsed by the European Medicines Agency and local government bodies [11–16]. People found to carry *DPYD* variants (c.1905 + 1G > A, (*DPYD* *2A), c.2846A > T, c.1679 T > G and c.1236G > A/HapB3), have decreased function of their alleles to make DPD enzyme, resulting in DPD deficiency and FP toxicity when standard FP doses are administered [17, 18] (Table 1). Approximately 8% of the European-Caucasian population carry one of these four variants, and other variants of significance in non-Caucasian populations are emerging and are likely to become incorporated in standard screening panels as supporting data expands [19, 20]. There is a limited understanding of *DPYD* expression in Aboriginal and Torres Strait Islander communities, although a small genomic study from a single indigenous population in the Tiwi Islands suggests that *DPYD* and *UGT1A1* expression differs from Caucasian data [21, 22].

**Table 1:** Clinically significant DPYD variants: variant allele frequency, DPD enzyme function, and relative-risk of FP-related toxicity

*DPYD* variant (rs number)	Variant Allele Frequency^a^ [3]	Allele function	Relative Risk of Toxicity at standard FP dose, (95% CI) [17, 18]
c.1905 + 1G > A, DPYD*2A (rs3918290)	0.9–1.5%	No function	2.85 (1.75–4.62)
c.1679 T > G (rs55886062)	0.1–0.2%	No function	4.40 (2.08–9.30)
c.2846A > T (rs67376798)	1.1–1.5%	Partial function	3.02 (2.22–4.10)
c.1236G > A/ HapB3 (rs56038477)	4.3–4.7%	Partial function	1.72 (1.22–2.42)

^a^European Caucasian data

Screening for *DPYD* variants prior to FP exposure allows an opportunity to dose-adjust FP chemotherapy regimens to make treatment more tolerable. Collaborative guidelines are in place to guide dosing decisions centred around which *DPYD* variants patients carry and tolerance of treatment, including those published by EviQ that guide dosing decisions in Australia [23]. While these guidelines do not mandate upfront *DPYD* screening, they support clinician-patient discussion and shared decision making.

A similar gene-drug relationship exists for uridine-diphosphate-glucuronosyltransferase family 1 (*UGT1A1*) gene and irinotecan chemotherapy. Irinotecan is typically prescribed in the management of pancreaticobiliary, other upper gastrointestinal, colorectal, sarcoma and lung cancers. Each year, approximately 2000 Australian cancer patients are prescribed irinotecan. Up to 40% of patients administered standard doses of irinotecan will develop grade 3–4 toxicities including diarrhoea and neutropenia [24]. Approximately 9% of European-Caucasians carry a *UGT1A1* variant; however, there is substantial ethnic variability throughout Asia (up to 41% variant carriers) [24]. There are 3 commonly significant variants; UGT1A1*6, *28 and *37, with *28 associated with the most significant functional deficit. Current guidelines recommend carriers homozygous for *UGT1A1* *28 and certain compound heterozygotes start with 70% of the normal irinotecan dose, but availability of supporting data outside of Europe is limited [24].

Pharmacogenomic (PGx) screening for *DPYD* variants has been embraced in Europe with positive data indicating improved patient safety and cost-effectiveness [17, 25, 26]. It is not yet known if a PGx screening intervention of this magnitude can be successfully and sustainably implemented in Australia. Consideration must be given to the geographical, institutional and ethnic differences in Australia as compared to Europe, including Aboriginal and Torres Strait Islander communities. To date, Australia has not been so forthcoming with support for an upfront screening strategy [8]. Glewis et al. (2024) report barriers to implementation, as described by Australian oncology clinicians, including the out-of-pocket (OOP) expense for patients and the perceived long turn-around time (TAT) of genotyping results [27]. Currently, Australian patients self-fund genotyping which excludes those without the means to afford testing.

Feasibility data from a pilot study (awaiting publication) conducted in regional Australia confirms a turnaround-time (TAT), on par with feasibility data from metropolitan institutions in the United Kingdom [28]. Furthermore, this pilot study evaluated clinician stakeholder perceptions of barriers and found similar themes to Glewis (2024), as well as lack of staffing and resources (education and support) [27]. An outstanding enabler within this pilot study was the degree of clinician motivation to improve the current system for the benefit of patient safety and delivery of innovative and high-quality healthcare.

Current gaps in knowledge regarding *DPYD* screening in Australia include:

- Will dose personalisation improve safety outcomes/ decrease toxicity for *DPYD* variant carriers who undergo dose personalisation in Australian practice?
- Is upfront *DPYD* screening cost-effective?
- What is the prevalence of the common 4 *DPYD* variants in the Australian population? Does this prevalence differ in non-Caucasian populations? Are there additional *DPYD* variants that are clinically significant that we must consider for an Australian-centric pharmacogenomic screening panel?
- Is *UGT1A1* genotyping feasible?
- What other factors can explain severe FP toxicity?

### Objectives and endpoints

GeneScreen 5-FU will confirm the evidence needed to successfully implement upfront *DPYD* PGx screening in Australia by demonstrating clinical impact, safety and cost-effectiveness. The primary objective is to establish an upfront PGx screening and dose modification pathway to guide dose personalisation and reduce FP-induced toxicity, improving patient safety. Secondary endpoints include confirmation of prevalence of *DPYD* variants in Australia, feasibility of genotyping, analysis of the cost-effectiveness of genetic screening and determination of cancer outcomes for *DPYD* carriers (Disease-Free Survival, DFS; Progression-Free Survival, PFS; Overall Survival, OS). Tertiary and translational endpoints will explore the feasibility of *UGT1A1* genotyping and explore the genomic traits of patients who develop severe toxicity in the absence of *DPYD* variants (and vice versa) (Table 2).

**Table 2:** GeneScreen 5-FU objectives and endpoints

**Objectives**	**Endpoints**
**Primary**	
Determine efficacy of upfront *DPYD* PGx-guided dose-adjustment on FP safety and toxicity of carriers of clinically significant *DPYD* variant alleles.	a) Frequency of serious FP-related toxicity in patients receiving FP chemotherapies: - Grade 3 and 4 toxicity (CTCAE v5), hospitalisations (including presentations not resulting in admission), ICU admissions and death (CTCAE v5, Grade 5). [5] - Case Report Forms (CRF) for toxicities Grade 3-5. b) Frequency of serious FP-related toxicity in *DPYD*variant carrier patientsreceiving FP chemotherapies: - Using same metrics as above.
Develop and implement strategies to address enablers and barriers identified in the feasibility study.	Using validated framework and including patient and clinician interviews [29, 30]
Determine cost-effectiveness of upfront* DPYD* genotyping and personalised dosing and estimate healthcare benefits of full implementation of *DPYD PGx*-guided dose-adjustment in an Australian population.	a) Health economic and cost-effectiveness analysis of upfront *DPYD* screening and PGx-guided dose-adjustments / individualisation. b) Apply health economic modelling to illustrate cost burden and cost-effectiveness of pre-emptive *DPYD* screening on health care services.
**Secondary Objectives**	
Determine feasibility of PGx screening and dose-adjustment.	a) Feasibility of *DPYD* genotyping and utility of dose recommendation guidelines: - TAT for genotyping. - Proportion of variant allele carriers who undergo dose-adjustment. Identification and exploration of adherence / deviations from dose-adjustment guidelines.
Confirm prevalence of clinically significant *DPYD *variants within the Australian community.	Measure frequency of each of the 4 *DPYD *variants c.1905+1G>A (rs3918290), c.2846A>T (rs67376798), c.1679T>G (rs55886062) and c.1236G>A (rs56038477).
Deliver a scientifically sound PGx protocol for pre-treatment genotyping that can be adopted into pathology laboratories across Australia.	Evaluate effectiveness of implementation research strategies using validated framework [29, 30]
[29, 30] Determine cancer outcomes for *DPYD* variant carriers with dose-adjusted FP compared to current standard of care.	Measure long-term outcomes including disease-free survival (DFS), progression-free survival (PFS) and overall survival (OS)
**Tertiary / Translational Objectives**^a^	
Identify *UGT1A1* variants (*6, *28 and *37) in patients receiving irinotecan and explore feasibility of expansion of a PGx panel to include *UGT1A1 *genotyping.	Determine *UGT1A1 *variant allele frequency and establish feasibility of genotyping pathway.
Explore other factors contributing to severe toxicity in patients not carrying the aforementioned *DPYD* variant alleles.	a) Consider genomic polymorphisms, within the *DPYD* gene or elsewhere, other downstream regulators and enzymes along the FP metabolic pathway to formulate translational investigations. b) Evaluation of DPD enzyme activity in selected cases: - Confirm that genotype correlates with phenotype measures (UH2/U concentration ratio, pharmacokinetics)

*CTCAE* Common terminology criteria for adverse events, *DFS* Disease-free survival, *DPYD* Dihydropyrimidine dehydrogenase (gene), *FP* Fluoropyrimidine, *OS* Overall survival, *PFS* Progression-free survival, *PGx* Pharmacogenomic, *TAT* Turn-around time, *UGT1A1* Uridine-diphosphate-glucuronosyltransferase family 1

^a^Translational endpoints only partially covered by current funding

## Methods/design

This is a single arm, non-randomised, multicentre prospective study. In recognition of the strong international data that show benefit for PGx-guided dosing, it is considered unethical for us to conduct a randomised controlled trial to directly compare the implementation and non- implementation of PGx-guided dosing. It is a collaborative, investigator-led initiative supported by a trial committee comprising researchers from most states in Australia.

### Study population

We aim to enrol 5000 patients from hospitals across multiple states in Australia and including metropolitan, regional and rural cancer services. Eligible participants are 18 years and older with solid organ malignancies intended to receive FP chemotherapies (either 5-FU or capecitabine) and/or irinotecan chemotherapy as part of either curative or palliative cancer management either as single agents or in combination. Patients must be able to provide informed consent and capable of providing a blood sample for genotyping. Exclusion criteria include those who have received prior treatment with FP chemotherapies, or who decline consent or blood collection. Patients who are pregnant or breastfeeding are also excluded, as are those already enrolled in other clinical trials that are likely to influence toxicity outcomes.

### Intervention

*DPYD* genotyping will be prospectively conducted in all patients prior to commencement of FP chemotherapies. Genotyping pathology services will be pre-determined by participating sites. Clinically significant *DPYD* variants will include c.1905 + 1G > A (rs3918290), c.2846A > T (rs67376798), c.1679 T > G (rs55886062) and c.1236G > A (rs56038477) (Supp Fig. 1). Patients found to carry one or more clinically significant variants will undergo dose-adjustment of FP chemotherapy prior to first administration, in accordance with eviQ guidelines (Supp Tables 1 and 2) [23].

- ◦ Heterozygote carriers of a single specified *DPYD* variant should receive a 50% dose-reduction prior to first FP exposure
- ◦ Following this administration, patients should be reassessed and FP dose further down-titrated or ceased in response to G3-4 toxicity, or up-titrated for those who experienced minor or no toxicities. Suggested increment for titration is ± 12.5% (of the 100% recommended dose), as suggested by EviQ.
- ◦ Patients who carry two variants (either compound heterozygote or homozygote carriers) should be dosed on an individual basis and may need to avoid FP administration altogether.

Data will be collected on all patients and will include specific datapoints for *DPYD* variant carriers who undergo dose-reductions to explore adherence to guidelines versus alternative dose-adjustment decisions. Bio-banked specimens from patients who do not have a pre-specified *DPYD* variant but experience ≥ G3 toxicities will undergo further exploratory genotyping. A subset of samples from patients intended to receive irinotecan chemotherapy will be batched and genotyped for clinically significant *UGT1A1* variants (*6 (*UGT1A1* c.211G > A), *28 A(TA)6TAA > A(TA)7TAA promoter), *37 (A(TA)6TAA > A(TA)8TAA promoter). Any *UGT1A1* genotype-guided dose-adjustment will be at clinician discretion. *UGT1A1* genotyping is for assessment of feasibility only. Data monitoring will be intermittently conducted by the trial management committee.

### Implementation sub-study intervention

Qualitative interviews will be conducted with a convenience sample of 20 to 40 patients and clinicians involved in PGx screening, with the sample size dependent on achieving sufficient breadth and depth as is favoured over saturation in this methodology [29]. Interviews with patients will be conducted following enrolment, genotyping and at least first cycle of chemotherapy. Clinicians will be contacted throughout the trial. This will be decided by the research team through discussions parallel to recruitment and data collection. Participants will be asked to complete semi-structured qualitative interviews over the telephone or teleconferencing which will be up to 45 min in length. Patient interviews will explore the degree to which people offered genotyping were aware of and understood the purpose of genetic testing and dose-personalisation in the trial, experiences of *DPYD* variant carriers who received *DPYD* genotype-guided dose-personalisation, including information needs around*,* and lived-experiences of dose-personalisation throughout treatment. Clinician interviews will explore the experiences of using *DPYD* PGx-guided dose-personalisation, including reasons for adjusting/not adjusting the dose, intention to continue using *DPYD* PGx-guided dose-personalisation, and barriers and facilitators to this prescribing method.

Interview recordings will be transcribed, deidentified and imported into a qualitative data analysis software package for analysis. Interview and focus group transcripts will be deidentified prior to analysis. Only members of the research team who are not involved in patient care will be involved in the analysis. Reflexive thematic analysis will be conducted to explore the commonalities and dissimilarities within and across the data, and reflexive practice will be employed to acknowledge and account for any subjectivities (bias) in the data analysis [30]. These findings will be used to optimise the PGx screening process and provide tailored support and educational resources.

### Clinical assessment, data collection and storage

The clinical assessment schedule is summarised below (Table 3), where implementation is included in the assessment table and is discussed separately below.

**Table 3:** GeneScreen 5-FU assessment schedule

Assessment Time-point	Data points collected	Comments
*Baseline*	Patient and cancer demographics FP chemotherapy data *DPYD* genotype status	FP containing regimens Chemotherapy indication
*Post-first FP dose*	Patient metrics FP dose and adjustments FP related toxicities Hospitalisations, ICU admissions, treatment-related death	G3-4 toxicities Time in hospital Time in ICU G5 toxicity-related deaths Dose reduction data
*Subsequent FP administration* *(capped at 60 days post-first dose)*	Patient metrics FP dose and adjustments FP related toxicities Hospitalisations, ICU admissions, treatment-related death	G3-4 toxicities Time in in hospital Time in ICU G5 toxicity-related deaths Dose reduction data
*No fixed time point*	Patient and clinician semi-structured interviews	Exploration of patient/clinician experience of genotyping process Clinician satisfaction with education/support resources, facilitators/barriers to ongoing use
*Follow-up*	Cancer outcomes reports at 6-month intervals up to 5 years post-FP	Disease-free survival (DFS) Progression-free survival (PFS) Overall survival (OS) All cause mortality

Initial assessment will include patient demographics, tumour data and chemotherapy indication (curative/palliative). Chemotherapy regimens and FP dosing will also be collected. Clinical parameters including height and weight and haematological results prior to each cycle will be recorded. Dates of *DPYD* sample collection and results will be recorded, as well as the variant carrier status (including *UGT1A1* where applicable).

Data from patients will be collected from clinical presentations within the first 60 days of their first FP dose to identify grade 3 and 4 toxicities requiring hospital presentation with or without admission, ICU admissions and deaths related to FP toxicities. Grading will be in accordance with the current CTCAEv5 [5]. For patients who carry *DPYD* variants and undergo dose-adjustments, FP dosing trends and adherence to/utility of the guidelines will be recorded. Patient follow-up data will be collected for 5 years to determine long-term cancer outcomes (DFS, PFS and OS). Data will be collected, de-identified and stored on a secure REDCap database. Patient samples will be de-identified and bio-banked.

#### Historical comparators

We felt it was unethical to have a control group of patients with *DPYD* variants who undergo standard FP dose administration due to the well described increased risk of toxicity within this population [9, 10]. Therefore, comparator populations for this project will include:

1. Current standard of care, as assessed by a retrospective review of 500 consecutive cases in NSW [31].
2. Contemporaneous cases without these *DPYD* variants treated with standard doses,
3. *DPYD* variant carriers in this study that do not receive PGx-guided dose-adjustment, and.
4. Patients treated in European cohorts whereby *DPYD* variant carriers were treated with a 25% or 50% FP dose reduction.

Background ≥ G3 toxicity in an ungenotyped population is approximately 17.4%, and in *DPYD* variant carriers without FP dose adjustment is reported to be 39–61% [8, 31]. Sub-analyses for each *DPYD* variant will be included.

### Statistical considerations

#### Statistical hypotheses

We hypothesise that FP-induced severe toxicity (≥ G3) will decrease from 60% in the variant carriers receiving full dose chemotherapy to 35% in variant carriers receiving PGx-guided chemotherapy.

#### Sample size determination

This project intends to capture and test most feasibly large cohort of patients based on average clinical volume and anticipated number of patients that are eligible for testing across all study sites. Approximately 17000 patients are treated with FP per annum, resulting in > 30,000 potentially eligible patients across just 2 years of recruitment. As such, 5000 subjects in this study would require a recruitment capacity of < 15%. Our participating sites provide care for > 30% of Australians with cancer.

Based on expected frequency of *DPYD* variant alleles (~ 4% as a conservative estimate), testing *N* = 5000 patients will result in ~ 200 patients with actionable genotypes. Toxicity in these patients will be compared to historical datasets (Table 4). Assuming toxicity decreases to 35% in those receiving PGx- guided dosing, *N* = 70/arm will provide 80% power to detect a significant improvement in FP-related toxicity at the 5% level.

**Table 4:** Populations for analysis

**Analysis Population**	**Description**
Testing Set	All patients who are eligible for recruitment and undergo* DPYD* genotyping
*DPYD *Set	Patients in this study who are found to carry a *DPYD *variant. Comparator groups include: 1) retrospective review of FP toxicity in *n*=500 patients who were treated without genotyping 2) contemporaneous cases without *DPYD *variants treated with standard doses 3) contemporaneous cases of *DPYD *variant carriers who do not receive dose-adjustment, and 4) *DPYD* variant carriers in European cohorts who were treated with dose reduction

### Statistical analyses

Descriptive statistics on continuous data will include means, medians, standard, deviations, and ranges, while categorical data will be summarized using frequency, counts and percentages. Graphical summaries of the data may also be presented.

The 95% confidence intervals for the proportion of tests returned within 7 days of intended chemotherapy dosing and the proportion of PGx guided dosing adjustments implemented will be estimated using the Clopper-Pearson method. PGx-guided dosing will be deemed feasible to implement if both 95% confidence intervals are completely above 80%.

The proportion of DPYD positive patients experiencing ≥ G3 toxicities in the current sample will be compared to the historical controls using a Fisher’s exact test. Logistic regression will also be used to compare the rate of ≥ G3 toxicities between the two groups. The intervention effect will therefore be summarised using the odds ratio and 95% CI from this model.

### Health economic analysis

A modelled cost-effectiveness analysis will estimate the incremental cost per QALY gained of *DPYD* screening. Prevalence, intervention costs and toxicity-related hospital costs (associated with inpatient separations, emergency department presentations and outpatient care) incurred during the study period will be used to populate a decision analytic model with a one-year time horizon. Health outcomes and hospital costs for a comparator group (i.e. no genotyping) will be estimated using published literature, analysis of a linked dataset (from historical cohorts) and prospectively in GeneScreen 5-FU participants that have a known *DPYD* genotype but did not have dose-adjustment. One-way sensitivity analyses will be conducted for key model parameters including *DPYD* screening costs, prevalence of *DPYD* genotypes and health outcomes. Probabilistic sensitivity analyses will be conducted to evaluate the impact of parameter uncertainty on model outcomes and will be presented on a Cost-Effectiveness Acceptability Curve for a range of cost-effectiveness thresholds. A budget impact analysis will be developed to inform on the affordability of national uptake of *DPYD* testing to support implementation.

### Implementation strategies

Semi-structured qualitative interviews of patients and clinicians will explore the attitudes and opinions to understand the experiences of those involved in *DPYD/UGT1A1* PGx screening. Forty representatives from patient and clinician groups will be selected at random. These data will be used to optimise the PGx screening process and provide tailored support and educational resources where required. Interviews with patients will be conducted following enrolment, genotyping and at least the first FP cycle. Clinicians will be contacted throughout the trial. Interviews will be recorded, de-identified and securely stored on password-protected university servers.

### Translational research

A portion of patients will develop ≥ G3 toxicities without being carriers of one of the genotyped *DPYD* variants, suggesting they either carry different clinically significant *DPYD* variant(s) or other genetic/epigenetic drivers of FP toxicity yet to be identified. We will conduct additional gene sequencing on bio-banked specimens to determine other genetic influences on FP toxicity. Furthermore, we will be able to provide further data regarding the limitations of linkage disequilibrium of c.1236G > A and HapB3 haplotype (c.1129-5923C > G, rs75017182) as described by Turner et al. [32]. This information will ideally help to tailor a PGx panel specific to our diverse Australian population to assist with identifying maximum numbers of patients at risk of toxicity prior to first FP exposure.

## Discussion

Although there have been many advances within pharmaceutical prescribing in oncology, fluoropyrimidines continue to form the backbone of many chemotherapy regimens used across a variety of solid organ cancers. With the rise of personalised and precision medicine it is imperative that the oncology community strive to make cancer therapies more tolerable for patients to facilitate adherence and efficacy, whilst considering the cost-effectiveness of these decisions on the wider health system. The implementation of upfront *DPYD* genotyping has been clinically and economically successful in countries where standardisation has been implemented [17, 25, 28].

Despite the incidence of avoidable serious FP-related toxicities and deaths, Australia is yet to adopt a standardised approach to minimise these complications. In additional to the perceived barriers held by clinical stakeholders, the expansive geographical reach of testing facilities and health services poses a unique barrier to the development of readily accessible PGx screening services with short TATs.

This research program intends to support the large-scale feasibility, safety and cost-effectiveness of upfront *DPYD* (and eventually *UGT1A1*) genotyping for people in Australia affected by cancer. Standardisation of PGx screening services and procedures across centres throughout multiple Australian states will help to create geographically local ‘hubs’ where genotyping can be offered and initiated in a time and cost-effective manner. Through the incorporation of implementation research strategies and patient and clinician feedback, this project intends to introduce a successful program that will maintain sustainability beyond the clinical trial environment. This program will create an accessible and equitable nation-wide PGx screening service available to all patients intended to receive FP chemotherapies. By improving personalised prescribing and limiting severe treatment-induced toxicity, patients will be more likely to complete intended treatment protocols and achieve better cancer related outcomes. Importantly, it will also serve as an Australian prototype not only for other gene/drug pairs implicated in oncology, but for other PGx screening programs across other health disciplines.

In addition, this study will develop an important database of patients who, despite “functional” *DPYD* genotypes, go on to develop ≥ G3 FP-induced toxicities. Further genomic analyses of these patients will help to uncover additional causative *DPYD* variants that may need to be considered for inclusion in a *DPYD* screening panel that is tailored to the Australian population. This includes understanding patterns of *DPYD* and *UGT1A1* expression in our Indigenous communities and working toward an inclusive pharmacogenomic screening panel. There may also be additional genetic and epigenetic factors identified within this Australian cohort that can be factored into the personalised prescribing of FP chemotherapies in the future.

## Supplementary Information

Supplementary Material 1.

Supplementary Material 2.

## Data Availability

No datasets were generated or analysed during the current study.

## Abbreviations

5-FU: 5-Fluorouracil
DFS: Disease-free survival
DPD: Dihydropyrimidine dehydrogenase (enzyme)
DPYD: Dihydropyrimidine dehydrogenase (gene)
FP: Fluoropyrimidine
ICU: Intensive care unit
OS: Overall survival
PFS: Progression-free survival
PGx: Pharmacogenomic
TAT: Turnaround-time
UGT1A1: Uridine-diphosphate-glucuronosyltransferase family 1
