# Supplementary material for: DPYD genotype-guided dose personalisation for fluoropyrimidine-based chemotherapy prescribing in solid organ cancer patients in Australia: GeneScreen 5-FU study protocol
Source: BMC Cancer. 2024 Nov 8;24:1369. doi: 10.1186/s12885-024-13122-8 (PMC11549825; doi:10.1186/s12885-024-13122-8)
Supplement: Supplementary file 1 — Supplementary Material 1. [file 12885_2024_13122_MOESM1_ESM.doc]

**GeneScreen 5-FU**

***DPYD* Genotype-guided dose Personalisation for Fluoropyrimidine prescribing in Cancer**

**Hunter Medical Research Institute, University of Newcastle, NSW**

**PARTICIPANT CONSENT FORM**

I, .......................................................................................................................[name]

have read and understand that the study will be conducted as described in the Information Statement, a copy of which I have retained.

I have been made aware of the procedures involved in the study, including any known or expected inconvenience, risk, discomfort or potential side effects and of their implications as far as they are currently known by the researchers.

I understand that my participation in this study will allow the researchers and others, as described in the Information Statement, to have access to my medical record, and I agree to this.

I agree to participate in this study and understand that I can withdraw at any time without providing a reason. I understand that my personal information will remain confidential to the researchers.

I have had the opportunity to have questions answered to my satisfaction.

I hereby agree to participate in this research study.

**NAME: _____________________________________________**

**SIGNATURE: _____________________________________________**

**DATE: _____________________________________________**

I agree to biobanking my specimen for use in future research: Yes No

I agree to be contacted about a research interview related to this study: Yes No

I would like a description of the research study outcome once complete: Yes No

**Declaration by person conducting the consent process**

**NAME: _____________________________________________**

**SIGNATURE: _____________________________________________**

**DATE: _____________________________________________**
