# Supplementary material for: DPYD genotype-guided dose personalisation for fluoropyrimidine-based chemotherapy prescribing in solid organ cancer patients in Australia: GeneScreen 5-FU study protocol
Source: BMC Cancer. 2024 Nov 8;24:1369. doi: 10.1186/s12885-024-13122-8 (PMC11549825; doi:10.1186/s12885-024-13122-8)
Supplement: Supplementary file 2 — Supplementary Material 2. [file 12885_2024_13122_MOESM2_ESM.docx]

**Supplementary Table 1. Dose recommendation for specific DPYD variants (from eviQ.org.au)(23)**

Recommendations in the tables below are based on the CPIC (2018) and DPWG (2020) guidelines, and in line with recommendations endorsed by the UK chemotherapy board (2020).

| Allele  (Heterozygous genotype) | Predicted % DPD enzyme activity  In the presence of one DPYD variant | Starting dose recommendation | Dose titration**  The dose increment (%) is a proportion of the target treatment dose (100%). |
| --- | --- | --- | --- |
| c.1905+1G>A  (IVS14+1G>A or rs3918290, also known as DPYD*2A) | 50% | 50% of target dose  or  consider alternative treatment*. | If tolerant after cycle 1, consider a dose increment of 12.5%.  Dose increments to a dose intensity of 75% of the target dose, over subsequent cycles may be possible. |
| c.1679T>G  (p.I560S or rs55886062,  also known as DPYD*13) | 50% | 50% of target dose  or  consider alternative treatment*. | If tolerant after cycle 1, consider a dose increment of 12.5%.  Dose increments to a dose intensity of 75% of the target dose, over subsequent cycles, may be possible. |
| c.2846A>T  (p.D949V or rs67376798) | 50 – 75% | 50% of target dose  or  consider alternative treatment*. | If tolerant after cycle 1, consider a dose increment of 12.5%.  Dose increments to a dose intensity of 75% of the target dose, over subsequent cycles may be possible.  If no toxicity is observed at a dose of 75%, a further increment to a maximum 85% may be possible, but caution is advised. |
| c.1236G>A/ HapB3  (rs56038477)  synonymous variant to  c.1129-5923C>G rs75017182 | 50 – 75% | 50% of target dose  or  consider alternative treatment*. | If tolerant after cycle 1, consider a dose increment of 12.5%.  Dose increments to a dose intensity of 75% of the target dose, over subsequent cycles may be possible.  If no toxicity is observed at a dose of 75%, a further increment to a maximum 85% may be possible, but caution is advised. |
| Treatment optimisation: | To maintain efficacy, consider increasing the dose after cycle 1 to a dose intensity of 62.5%, in patients who experience no or clinically tolerable toxicity. Where treatment cycles are defined as Monday to Friday (over 5 days), it may be recommended to increase doses at a later stage.  Consider conservative dose increments in patients who tested positive for c.1905+1G>A and c.1679T>G, as these two variants have the most deleterious effect on DPD enzyme activity.  To minimise toxicity-related complications, decrease the dose or withhold treatment promptly in patients who experience clinically intolerable toxicity. | | |

* When alternative therapy is recommended the therapy is to be determined by the treating oncologist, and informed by the tumour type, clinical indication and predicted severity of enzyme deficiency based on DPYD genotype. It is beyond the scope of this guideline to state the appropriate decision making for each indication.

** In rare cases, patients may tolerate a dose intensity of near 100% of the target treatment dose.

**Supplementary Table 2. Dose recommendation for more than one DPYD variants (eviQ.org.au)**

| Genotypes  Homozygous (two of the same variants)  Or  Compound heterozygous (two different variants) | Predicted % DPD enzyme activity  In the presence of two DPYD variants | Starting dose recommendation | Dose titration |
| --- | --- | --- | --- |
| c.1905+1G>A and c.1905+1G>A  homozygous  or  c.1679T>G and c.1679T>G  homozygous  or  c.1905+1G>A and c.1679T>G  compound heterozygous | 0%  Complete DPD deficiency | DO NOT administer fluoropyrimidine therapy in patients with these genotypes. | Not applicable |
| c.1905+1G>A and c.1236G>A/HapB3  compound heterozygous  or  c.1905+1G>A and c.2846A>T  compound heterozygous  or  c.1679T>G and c.1236G>A/HapB3  compound heterozygous  or  c.1679T>G and c.2846A>T  compound heterozygous | 10 – 25% | Consider alternative treatment*.  Where alternative therapy is not considered suitable, 5-FU (IV) may be considered in centres with expertise and therapeutic drug monitoring (TDM) services. Consider a strongly reduced dose at 10% of the target dose. | If tolerant after cycle 1, titrate dose against toxicity over subsequent cycles to a maximum of 25% of the target dose. |
| c.1236G>A/HapB3 and c.1236G>A/HapB3  homozygous  or  c.2846A>T and c.2846A>T  homozygous  or  c.1236G>A/HapB3 and c.2846A>T  compound heterozygous | 10 – 50% | Consider alternative treatment*.  Where alternative therapy is not suitable, 5-FU (IV) may be considered in centres with expertise and therapeutic drug monitoring (TDM) services. Consider a strongly reduced dose at 10% of the target dose. | If tolerant after cycle 1, titrate dose against toxicity over subsequent cycles to a maximum of 50% of the target dose. |
| Treatment optimisation: | In patients who experience severe toxicity, promptly decrease the dose or withhold treatment until toxicity has resolved.  Centres with TDM services, are recommended to utilise TDM to determine plasma 5-FU concentrations at the earliest timepoint of steady state, and to cease treatment if concentrations are too high. | | |

* When alternative therapy is recommended the therapy is to be determined by the treating oncologist, and informed by the tumour type, clinical indication and predicted severity of enzyme deficiency based on DPYD genotype. It is beyond the scope of this guideline to state the appropriate decision making for each indication.
